# Adjunctive IlluminOss Fixation for a Vancouver B1 Periprosthetic Femoral Fracture: A Case Report

**DOI:** 10.7759/cureus.107639

**Published:** 2026-04-24

**Authors:** Ahmad Ismail, Wayne Ngo, Niladri Basu

**Affiliations:** 1 Medicine, University of Texas at Arlington, Arlington, USA; 2 Radiology, University of Arkansas for Medical Sciences, Little Rock, USA; 3 Orthopedics, Dallas Orthopaedic Trauma Institute, Dallas, USA

**Keywords:** distal femoral fracture, illuminoss implant, minimally invasive fixation, periprosthetic femur fracture, trauma and orthopedics, vancouver fracture types

## Abstract

Open reduction and internal fixation with plates and intramedullary nails is often the standard approach for periprosthetic femoral shaft fractures. However, in patients with recent hip arthroplasty, these traditional methods may be invasive, difficult to position, or carry a higher risk of periprosthetic complications. The IlluminOss system (IlluminOss Medical, East Providence, RI, USA) offers a minimally invasive alternative that delivers a light-curable polymer through a balloon catheter that takes the shape of the intramedullary canal without the need for rigid instrumentation that may induce intraoperative trauma. A 63-year-old female with a recent right total hip arthroplasty presented with right thigh pain following a ground-level fall. Imaging revealed a Vancouver B1 periprosthetic fracture of the distal femoral diaphysis extending proximally toward the femoral stem, which was treated with an intramedullary IlluminOss polymer implant supplemented with lateral plate fixation. This case highlights the use of a light-curable polymer implant as a minimally invasive adjunct to plate fixation for periprosthetic femoral fractures.

## Introduction

Fractures occurring near prior surgical sites exhibit reoperation rates of up to 20%, largely due to compromised bone quality in these areas [1,2]. This elevated risk limits the operative methods available for fixation. Additionally, periprosthetic fractures can further complicate operative methods due to space constraints. The IlluminOss implant (lluminOss Medical, East Providence, RI, USA) offers a minimally invasive solution that reduces intraoperative trauma while providing sufficient stabilization to support proper healing. In this case report, we discuss the application of IlluminOss for a periprosthetic distal femur fracture and compare it to contemporary options [3,4].

## Case presentation

**Figure 1:**
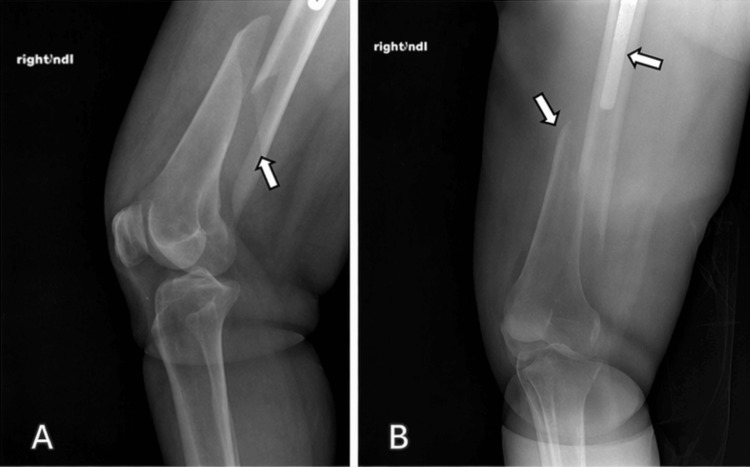
Anteroposterior (A) and lateral (B) preoperative radiograph demonstrating a displaced spiral fracture involving the distal femoral diaphysis consistent with Vancouver B1 periprosthetic fracture classification

A 63-year-old female with a past medical history of premature ventricular contractions, shoulder arthroplasty, and Raynaud’s syndrome presented to the emergency room with an oblique fracture of the distal femoral diaphysis, with the fracture line extending proximally up to the distal aspect of the femoral stem (Figure 1). Preoperative planning was made to utilize the IlluminOss implant due to its customizable length, drillable abilities, and minimally invasive extramedullary support capabilities.

During the operation, the patient was positioned supine on the operating table. A standard lateral approach was used for open reduction and internal fixation (ORIF) with plate fixation and mini-fragment fixation. A lateral corticectomy was performed using a 3-mm drill bit through the lateral femoral metaphyseal cortex. A guidewire was then placed into the remaining femoral shaft and sequentially reamed up to 9 mm.

**Figure 2:**
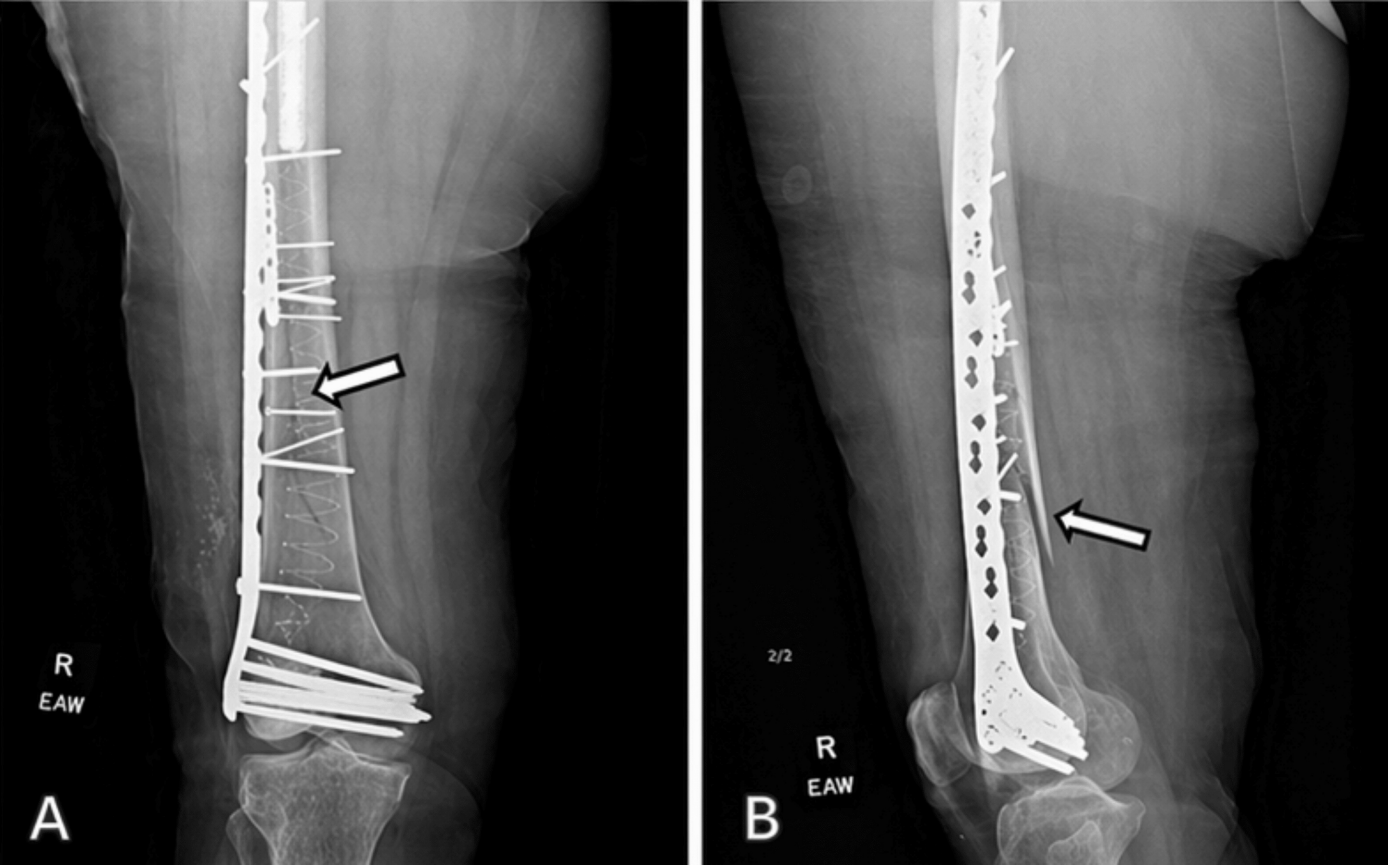
Immediate postoperative radiograph demonstrating IlluminOss intramedullary implantation with subsequent plate and screw fixation The fracture is well reduced with good anatomical alignment.

An IlluminOss balloon catheter was subsequently inserted and filled with a proprietary monomer solution, which was allowed to polymerize, forming an intramedullary support structure. Once polymerization was complete, the balloon catheter was detached and removed, leaving the hardened polymer implant. Following polymerization, a Synthes VA-LCP curved condylar plate (Synthes GmbH, Oberdorf, Switzerland) was applied laterally to span the fixation construct and secured with multiple screws to enhance distal fixation and provide structural support. Immediate postoperative radiographs demonstrated plate-and-screw fixation, satisfactory reduction, and maintained alignment (Figure 2).

**Figure 3:**
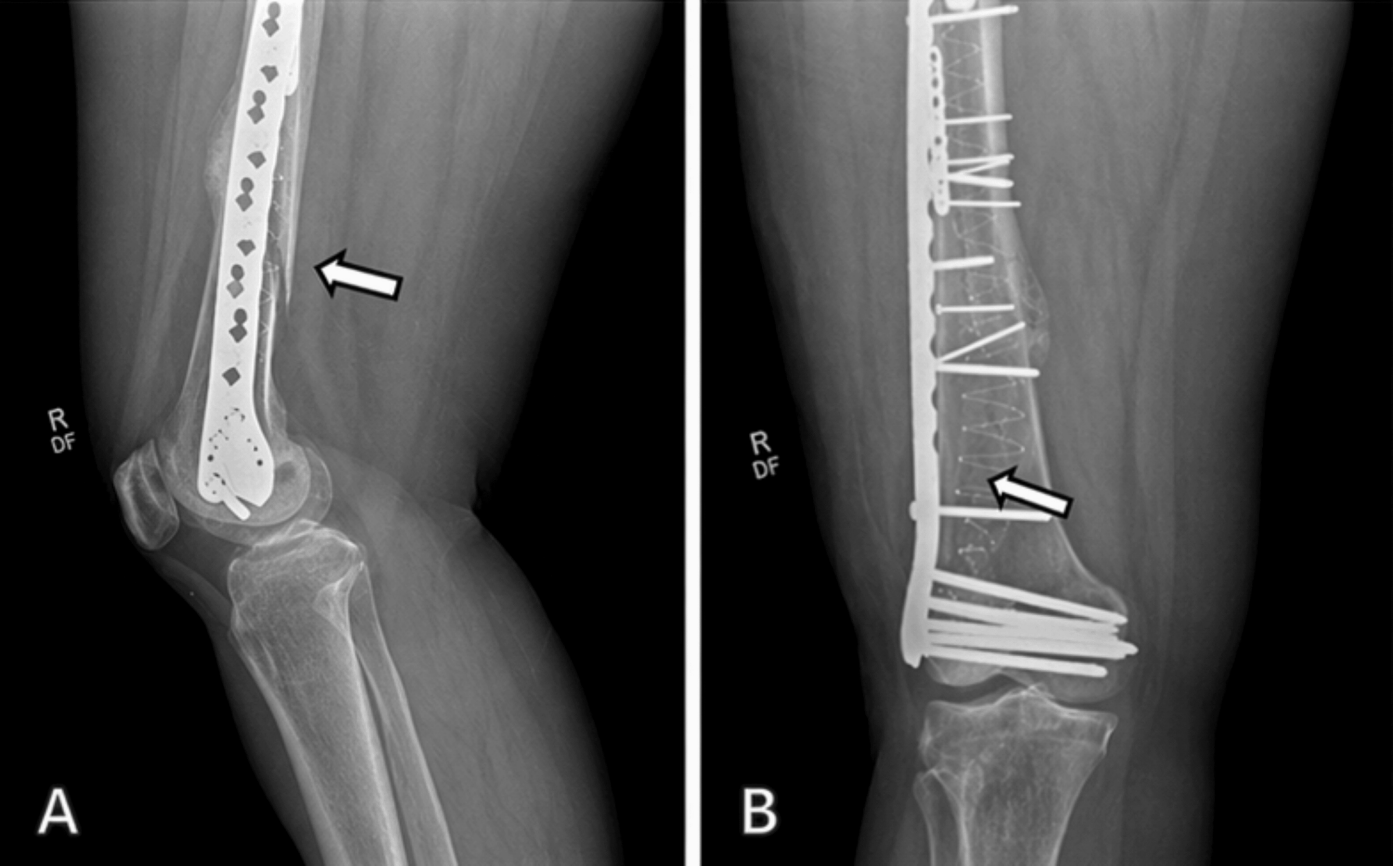
Three-month postoperative imaging demonstrating maintained alignment and stable fixation

**Figure 4:**
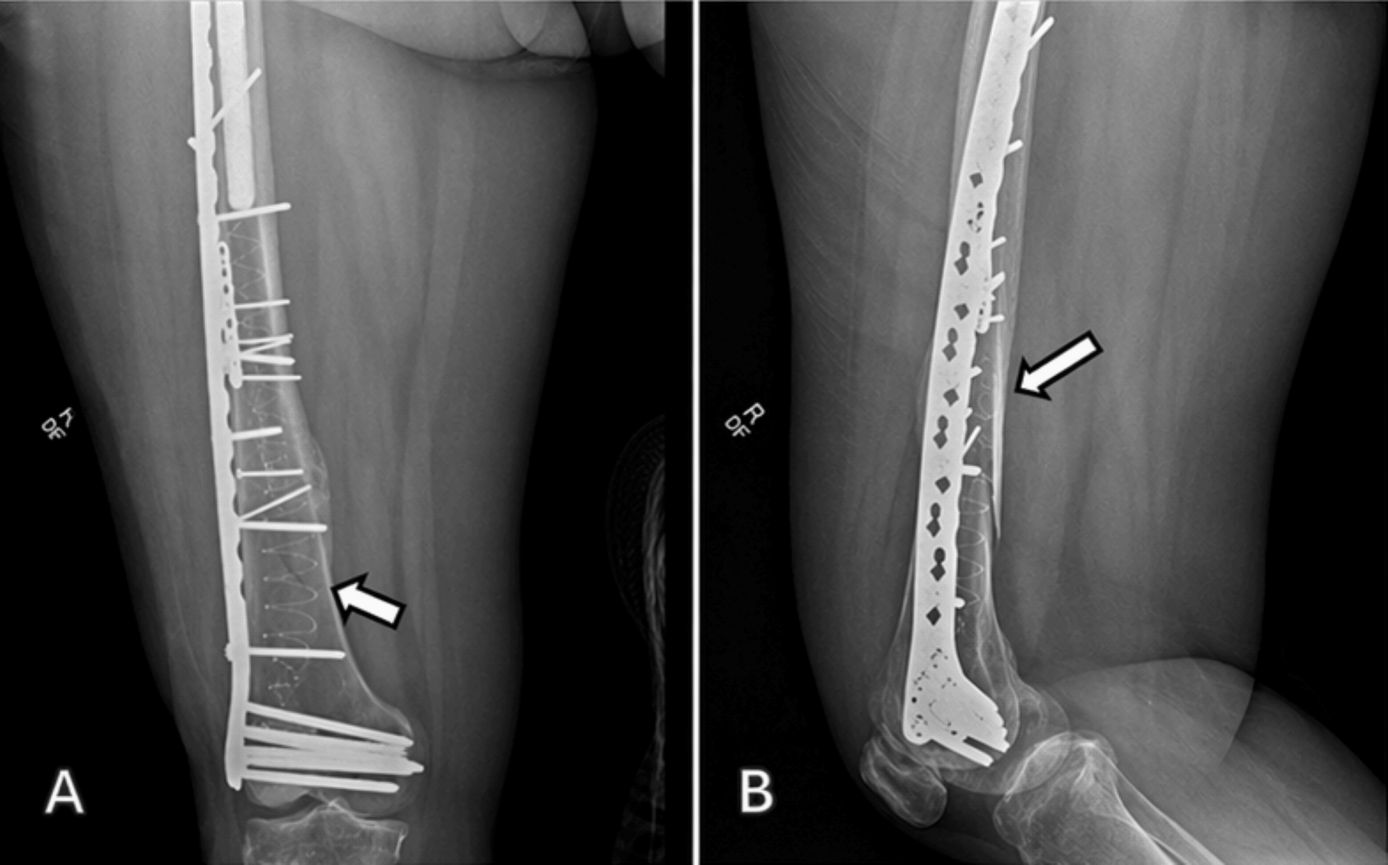
Six-month follow-up radiograph demonstrating a well-fixed and well-positioned construct with continued fracture healing No evidence of hardware failure or loosening.

The postoperative course was uncomplicated. The patient was maintained on toe-touch weight-bearing for six weeks, followed by progression to weight-bearing as tolerated. At three-month follow-up, radiographs demonstrated maintained alignment and stable fixation (Figure 3). At six-month follow-up, radiographs demonstrated continued fracture healing with stable hardware and no evidence of loosening or fixation failure (Figure 4). The patient also reported improvement in pain and functional mobility.

## Discussion

Periprosthetic femur fractures after total hip replacement can be difficult to manage, especially when bone quality is poor. These fractures are usually treated with locking plates, dual plating, or revision surgery depending on the stability of the implant [5]. Additional reported treatment options for Vancouver B periprosthetic femoral fractures include ORIF, cementless modular or monoblock long-stem revision, cement-in-cement revision, cemented long-stem revision, and impaction bone grafting. The choice of treatment depends on a combination of implant stability, fracture characteristics, patient factors, and surgeon judgment [6]. Dual plating, a commonly used technique, has shown highly favorable clinical outcomes in implant-stable fractures, with one series reporting union in 24 of 26 patients (92%) with minimum follow-up, although there is no clear consensus on optimal treatment [7].

These approaches often require larger surgical exposure and can disrupt surrounding soft tissue and blood supply [5,8]. IlluminOss has been more commonly used in fractures related to metastatic bone disease and osteoporosis due to its ability to create internal support with less surgical disruption and to provide a custom prosthetic fit, which other techniques may not offer [9,10]. Because it does not require rigid metal instrumentation inside the canal, it may reduce soft tissue trauma compared to traditional techniques. Clinical use of IlluminOss to treat femoral fractures in humans remains limited; however, preclinical data from a rabbit study demonstrating femoral repair showed successful bone healing and callus remodeling over six months [11].

In this case, the patient had a recent total hip replacement, and care was taken to avoid disturbing the existing implant. The IlluminOss implant provided internal support, while a lateral plate added stability. This approach allowed for fixation without entering the knee joint or interfering with the femoral stem. This case suggests IlluminOss may be a useful option in selected patients beyond its reported use in osteoporosis and metastatic lesions. Additional studies are needed to better understand its role in traumatic fracture management.

## Conclusions

This case demonstrates that the IlluminOss light-curable polymer implant can be a safe and feasible adjunct to traditional fixation methods for periprosthetic femoral fractures in selected patients, especially those with recent joint replacements and concerns for poor bone quality. Compared to dual plating and locking compression plates, IlluminOss may minimize periosteal and soft tissue disruption due to its minimally invasive design. The system allows for customizable implant sizing and extra-articular access, avoiding violation of the knee joint. Its intramedullary, drillable polymer structure provides internal support while permitting additional fixation to enhance stability. In this case, the minimally invasive approach resulted in stable fixation with appropriate early fracture healing and improved functional outcomes. IlluminOss may be a useful alternative in select complex cases where standard fixation techniques carry increased risk.
